# Long-term environmental background radiation is associated with urinary tract cancer incidence: A population-based study from Finland

**DOI:** 10.1016/j.canep.2025.102912

**Published:** 2025-09-10

**Authors:** Peng Li, Mikko Myrskylä, Pekka Martikainen

**Affiliations:** a Max Planck Institute for Demographic Research, Rostock, Mecklenburg-Vorpommern, Germany; b Max Planck – University of Helsinki Center for Social Inequalities in Population Health, Rostock, Germany and Helsinki, Finland; c Helsinki Institute for Demography and Population Health, University of Helsinki, Helsinki, Finland

**Keywords:** Environmental, Radiation, Radon, Uranium, Urinary tract cancer, Kidney, Bladder

## Abstract

**Background:**

The long-term carcinogenic effects of natural radioactive elements in the environmental background on urinary tract cancer (UTC) have not been fully investigated in the general population.

**Methods:**

The entire Finnish population was linked to long-term municipality-level data on concentrations of uranium and radon in water and radon in indoor air by residential location annually between 1987 and 2016, and UTC incidence was tracked until 2021 based on multiple registries. The first principal component (PC1) of the three radiation exposures was used as a proxy for radiation exposure, and was assessed using the Cox proportional hazards model for its association with UTC risk. Age-standardized incidence rates (ASR) and population attributable fractions (PAF) of radiation exposure were estimated. Spatial association between regional radiation exposure and UTC risk was assessed.

**Results:**

Among a total of 2,816,495 residents with 30-year exposure data, 6718 primary UTC cases were diagnosed during 2017–2021. UTC risk increased significantly with each standard deviation (SD) increase in PC1 (hazard ratio [HR] = 1.05, 95 %CI 1.02–1.07). Dose-response relationship was observed when exposure was modelled via natural cubic splines. Increasing UTC incidence was observed across exposure quintiles (lowest quintile: ASR = 47, 95 % CI 45–50; highest quintile: ASR = 53, 95 %CI 50–55). About 5.1 % (PAF, 95 %CI 0.5 %–9.7 %) of UTC incidence was attributable to radiation exposure. Stronger association (HR=1.06, 95 %CI 1.03–1.09, per 1-SD increase) and attributable fraction (PAF = 9.0 %, 95 %CI 3.2 %–14.8 %) were observed among men than among women. Regional UTC risk was significantly associated with regional radiation exposure adjusted for the spatial lag effect in the total population (*β* = 0.08, *p* < 0.01), men (*β* = 0.07, *p* < 0.01) and women (*β* = 0.1, *p* = 0.02).

**Conclusion:**

Association between long-term environmental radiation exposure and UTC risk was observed in the Finnish population. Dose-response patterns were observed, especially among men. UTC risks attributable to radiation exposure and geographical patterns warrant further investigations.

## 1. Introduction

In 2020, over one million urinary tract cancers (UTC), including 431,000 kidney cancer and 573,000 urinary bladder cancer cases, were diagnosed globally [1]. The common risk factors related to kidney and bladder cancer are age, sex (men have a higher risk), obesity, smoking, hypertension, family history of cancer, genetic susceptibilities, and environmental exposures [2-5]. The kidney and bladder cancer incidences and mortality rates are higher in European countries than in developing countries [6]. A strong but unclear geographical pattern of kidney cancer incidence has been observed in European countries, with Central and Northern Europe having particularly high incidence rates [7,8]. This pattern is coincident with the geographical patterns of natural radioactivity, with higher radiation levels being observed in Central and Northern Europe, including in countries such as the Czech Republic and Finland [9]. However, the association between these two phenomena has not been fully investigated.

Radionuclides from the environment, including uranium and radon, have been classified as Group 1 human carcinogens by the WHO [10]. Positive associations between radioactivity and kidney or bladder cancer mortality have been reported in uranium miners and radiation workers, in atomic bomb survivors, and in the general population [11-16]. However, evidence challenging these findings has been reported by other studies examining uranium miners and radiation workers [17-23] and the general population [18,24-26]. The discrepancies between previous studies might be related to the accuracy of exposure metrics, confounding risk factors, and limited sample sizes [27]. Larger general population studies using long-term and accurate metrices are needed to clarify the relationship between radiation exposure and UTCs.

In this paper, we tracked the dwelling information of the general Finnish population and linked it to long-term data on environmental radioactivity. As measuring low-dose environmental radiation exposure is challenging, we used long-term municipality-level data on concentrations of uranium and radon in borehole water and of radon in indoor air as proxies for environmental radioactivity. The associations between 30-year average radiation exposures and UTC (kidney or bladder cancer) risks were investigated in the total sample and stratified by sex. The age-standardized incidence rate (ASR) and the population attributable fraction (PAF) of radiation exposure were estimated. The correlations between geographical patterns of UTC risks and regional radiation levels were assessed.

## 2. Materials and methods

### 2.1. Population and study design

**Fig. 1.**
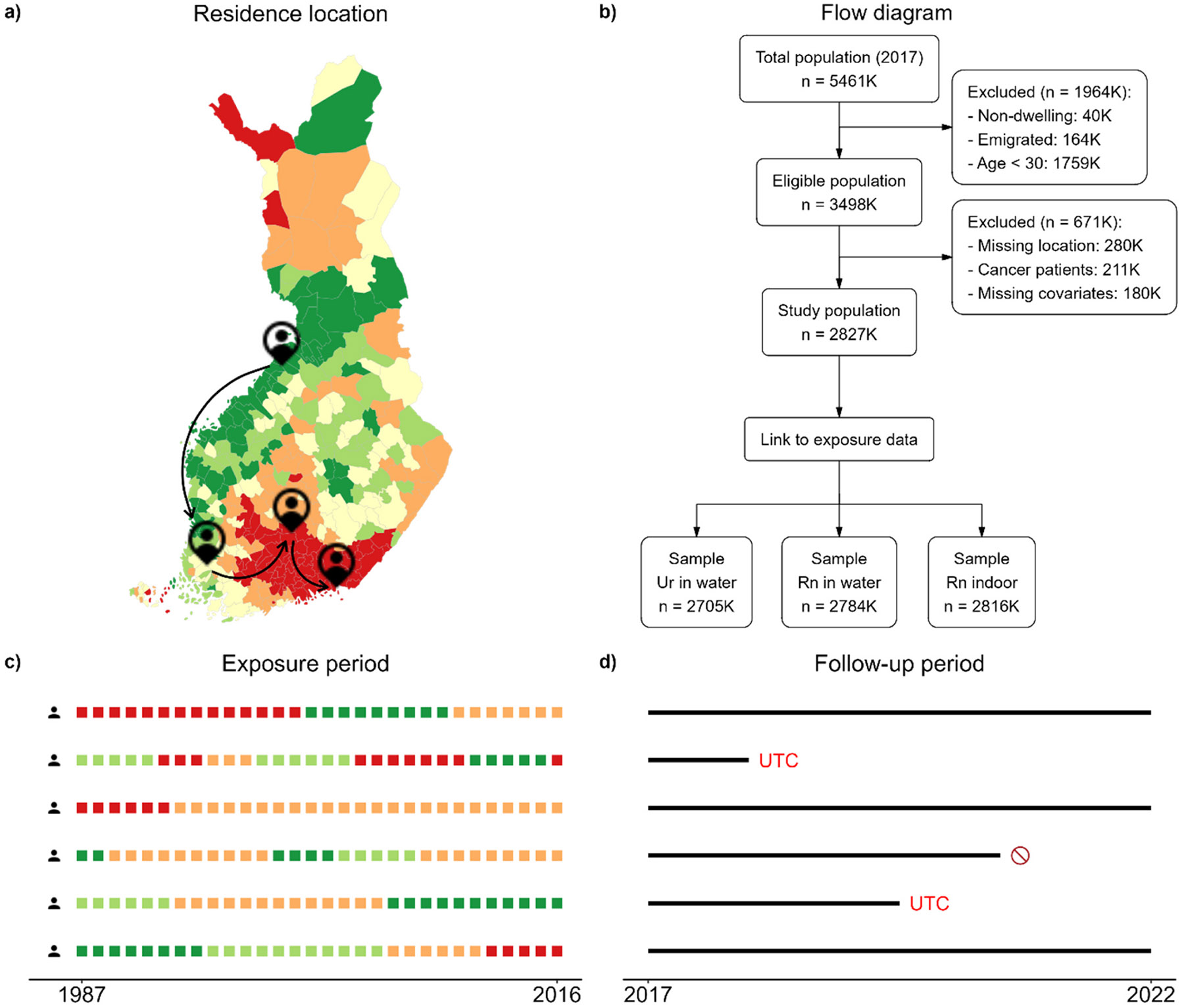
Study design and flow diagram, a): The annual residential location was recorded for each individual. b): Of the total population of 5.5 million, individuals who were under age 30 or had emigrated at baseline (January 1, 2017) were excluded. Of the eligible population of 3.5 million, about 0.7 million individuals were excluded because they had missing residential location information, a cancer diagnosis before 2017, or missing information on the socio-demographic covariates. A total of 3.7 million residents were included in the study, of whom about 28 million had 30 years of exposure data. c): The exposure period lasted from 1987 until the end of 2016. The residential locations and regional radiation exposures at the municipality level were recorded and the average exposure levels were calculated. d): All subjects were tracked for UTC diagnosis over the follow-up period lasting from the start of 2017 until the end of 2021. Individuals who died or were diagnosed with other cancers during the follow-up period were censored.

This study was based on multiple national registries in Finland covering the total population living in Finland from 1987 until 2021. The entire Finnish population who were residing continuously in Finland from 1987 to 2016 (exposure period) and had reached age 30 or older on January 1, 2017 (baseline) were investigated. Samples with different race backgrounds, including minorities and immigrants, were included in the study. Each individual’s (municipality-level) residential location in each year was tracked from 1987 until 2016 based on register data of Statistics Finland, and was linked to municipality-level radiation concentrations (Fig. 1, panel a). Average exposures were calculated based on these 30-year records for each individual (Fig. 1, panel c). Individuals who had less than 30 years of residential location data, were diagnosed with cancer before the baseline, or were missing important covariates were excluded. Individuals were followed from the baseline until a diagnosis of primary urinary tract cancer (ICD-O 3 kidney: C64–C66, C68; bladder: C67) using the Finnish National Cancer Register (1953–2021), a diagnosis of another type of cancer, emigration, death, or December 31, 2021, whichever happened first (Fig. 1, panel d). The flow diagram of the study design is shown in (Fig. 1, panel b).

### 2.2. Environmental radiation exposures

Municipality-level radon and uranium concentrations in borehole water were reported in the Finnish Borehole Water Radon and Uranium Atlas (2012) by the Radiation and Nuclear Safety Authority (STUK), Finland [28]. The concentrations of radon were based on measurements from the late 1960s to July 2012 in approximately 11,300 boreholes connected to a permanent residence. These measurements covered about 10 % of boreholes in 296 municipalities. The concentrations of uranium were based on measurements from the late 1970s to July 2012 in approximately 5700 boreholes connected to a permanent residence in 258 municipalities. Data on municipality-level radon concentrations in indoor air were released as a radon map by STUK [29,30]. The indoor air radon concentrations were measured based on STUK’s radon measurement boxes in low-rise residential buildings from 1986 until 2020. The average value of two or more boxes measured simultaneously, or the maximum value of measurements carried out at different times, was used to calculate annual average radon concentration in the dwelling, which was obtained by multiplying the result of the measurement made during the measurement period (from the beginning of September to the end of May) by 0.9. Radon mitigation effects for measurement time and buildings constructed in different years were adjusted in calculating municipality-level radon concentrations by STUK [29,30]. Data on median radon concentrations in indoor air for former municipalities (administrative changes) were extracted from the Radon Atlas of Finland (2010) [31]. The percentage of measured dwellings within each municipality was also extracted from the Radon Atlas. A total of 320 municipalities had available data on radon concentrations in indoor air. A total of 79 municipalities with more measurements on three exposures were defined as regions with high data quality (HQ region) for sensitivity analyses. The criteria were: with more than 5 measurements for uranium in water; with more than 15 measurements for radon in water; and with more than 100 measurements which covered no less than 5 % of total residential buildings within the municipality for radon in indoor air.

### 2.3. Definition of key covariates

Individual socio-demographic characteristics at baseline, including birth date, death date, educational level, income, marital status, and residence information, were extracted from Statistics Finland. The birth cohorts were classified into five-year groups. Educational level was defined according to the International Standard Classification of Education (ISCED-2011) as low (ISCED 0–2), medium (ISCED 3–4), or high (ISCED 5–8). Income measured as disposable personal income was based on salary, self-employment, and property income and current transfers received after taxes, and was classified as low, medium, or high based on terciles within five-year age groups. Marital status was classified as married, unmarried, divorced, or widowed. Information on the urbanisation level (urban, semi-urban, rural) of the municipality of residence and the living conditions (house or apartment) for each individual from 1987 until 2016 was also extracted. Years lived in an urban/semi-urban/rural region and years lived in a house during the exposure period were calculated for each individual.

Municipality-level lung cancer incidence rates from 1988 until 2021 were calculated based on Finnish National Cancer Register data using the European standard population (2013 edition) as the reference [32]. Individual-level health conditions before the baseline were extracted from the Finnish Social Insurance Institution’s Prescription Register (1995–2021) and the National Institute of Health and Welfare Registry (1970–2021). The health conditions of diabetes (ATC code A10; ICD-9 code 250; ICD-10 code E08–E13), hypertension (ATC code C02; ICD-9 code 401; ICD-10 code I10), and chronic kidney disease (ATC code C03; ICD-9 code 585; ICD-10 code N18) were defined based on the recodes in these two registries. The health conditions of obesity (ATC code A08; ICD-9 code 278; ICD-10 code E66), calculus in kidney (ICD-9 code 592.0; ICD-10 code N20), calculus in lower urinary tract (ICD-9 code 594; ICD-10 code N21), and urinary tract infections (ICD-9 code 590, 595 and 599; ICD-10 code N10, N30 and N39) were based on the records for the five-year period before the baseline. Family history of cancer was identified in parents and biological siblings based on the cancer registry and child-parents registry (1857–2021).

### 2.4. Statistical methods

Baseline socio-demographic characteristics, radiation exposure levels, health conditions, and cancer diagnostic characteristics were summarised. Municipality-level average radioactivity concentrations were shown in maps, and distributions of individual exposures were shown in histograms. The three 30-year average radiation exposures of the total population were summarized using principal components (PC1, PC2, and PC3). The first component (PC1) was used as a proxy for individual environmental radiation exposures (Supplementary Figure S3). Cox proportional hazards regression models using age as the time scale were used to investigate the risk of UTC for every one standard deviation (SD) increase in PC1. To investigate dose-response relationships between exposures and UTC risks, the exposure component PC1 was fitted as a continuous variable using natural cubic splines. PC2, PC3, and sex were adjusted in the baseline model (Model 1). Education, income, marital status, years lived in a house, years lived in an urban region, municipality-level lung cancer incidence rates, family history of cancer and health conditions (diabetes, hypertension, obesity, chronic kidney disease, calculus in kidney, calculus in lower urinary tract, and urinary tract infections) were further adjusted in Model 2. To eliminate potential heterogeneities from different birth cohorts, the main model (Model 3) was further conditioned on five-year birth cohorts. The proportional hazards assumption was assessed using the log(-log) approach and plots of Schoenfeld’s residuals.

In sensitivity analyses considering the disparities in radiation exposure between people living in urban and rural areas, the same analyses were performed stratified by years lived in an urbanized region (*<*15 vs ≥15 years) during the exposure period. Considering the quality of radiation exposure measurement in different regions, the same analyses were performed on samples who lived in an HQ region ≥ 10 years during the exposure period. All the analyses were performed on the total population and stratified by sex. The same analyses were performed to assess the effect of radiation exposure on kidney cancer risks and bladder cancer risks. To control for multiple comparisons, results with false-discovery rate (FDR) < 0.05 were highlighted.

To investigate the absolute effect of radiation exposure on UTC risk, the PC1 was classified into quintile levels. Age-standardized incidence rates (ASRs) of UTC per 100,000 person-years based on the European standard population (2013 edition) were calculated for each quintile level. The population attributable fraction (PAF) of radiation exposure was estimated for the total population, for the population who mainly (≥10 years) lived in an HQ region, and for men and women separately.

The regional radiation exposures were defined as the average value of the first principal component (PC1) of municipality-level radiation exposures for municipalities within each region (Supplementary Figure S3). The regional UTC risks were estimated via the Cox model for 19 residential regions at baseline using Uusimaa as the reference region. The association between regional radiation exposures and regional UTC risks was assessed using a linear spatial lag regression model.

All analyses were conducted using the statistical software R version 4.3.1 [33].

## 3. Results

A total of 2,816,495 individuals residing in Finland were included in the study. The average age of the participants at the baseline date of January 1, 2017 was 60.1 (standard deviation, SD = 12.2). A total of 6718 UTC cases were diagnosed (4462 kidney cancer cases and 2256 bladder cancer cases) in the follow-up period. The average exposure levels were 24.8 (natural log-scale: mean=3.2, SD=1.5) μg/L for uranium in borehole water, 502.7 (natural log-scale: mean=6.2, SD=1.05) Bq/L for radon in borehole water, and 90.9 (natural log-scale: mean = 4.5, SD = 0.46) Bq/m3 for radon in indoor air (Table 1). Municipality-level radiation concentrations were shown as maps and histogram plots in Supplementary Figure S1. The numbers of observations of the three radiation exposures were shown as maps and histogram plots in Supplementary Figure S2. Correlations between the three radiation exposures and the results of the principal components analysis were shown in Supplementary Figure S3. Histogram plots of individual-level 30-year average exposures were shown in Supplementary Figure S4. Sample characteristics by radiation exposure (PC1) quintile levels were shown in Supplementary Table S1.

### 3.1. UTC risks and radiation exposures

#### 3.1.1. Main analyses

**Fig. 2.**
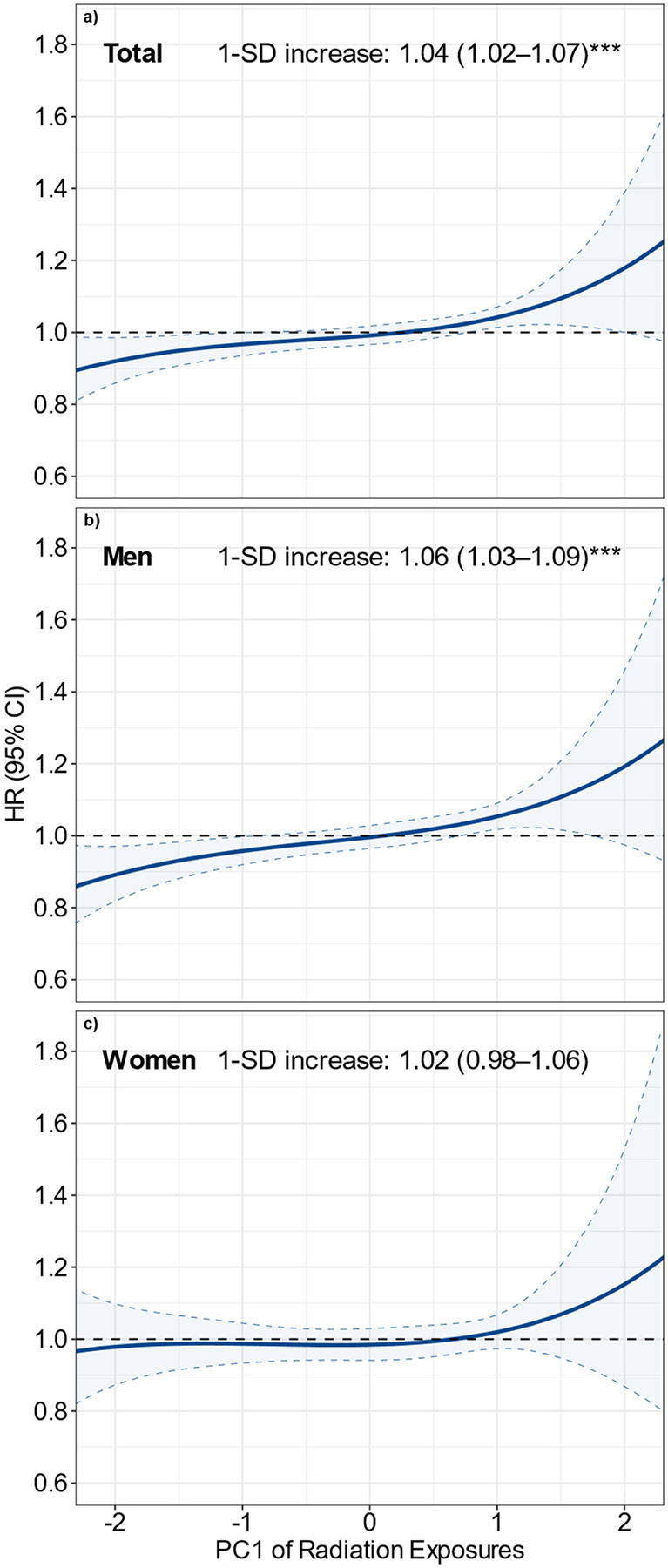
Association between UTC incidence and the first principal component (PC1) of the three exposures modelled with natural cubic spline in the total population and stratified by sex, Hazard ratio (HR, blue line) and 95 % confidence interval (CI, shaded area in light blue) of UTC incidence modelled with natural cubic spline of the first principal component (PC1) of the three radiation exposures. The Cox proportional hazards regression model (Model 3) conditional on five-year birth cohorts using age as the time scale was adjusted for the other two principal components of radiation exposures, sex (for the total population), individual educational level, income, marital status, years lived in a house, years lived in an urban region, municipality-level lung cancer incidence rates, and family history of cancer and health conditions (diabetes, hypertension, obesity, chronic kidney disease, calculus in kidney, calculus in lower urinary tract, and urinary tract infections) at baseline. The samples were further stratified by sex. HRs and 95 % CIs of UTC incidences associated with per SD increase of PC1 (as a continuous variable) were annotated. † *p*-value < 0.1; * *p*-value < 0.05; ** *p*-value < 0.01; *** *p*-value < 0.001.

In the total population, elevated UTC risk was significantly associated with higher radiation exposure (PC1) (hazard ratio [HR] with one SD increase in PC1 1.05, 95 % CI 1.02–1.08), while radiation exposure (PC1) was fitted as a continuous variable. Among men, elevated UTC risk was significantly associated with higher radiation exposure (HR 1.06, 95 %CI 1.03–1.09). Among women, elevated UTC risk was positively but non-significantly associated with higher radiation exposure (HR 1.02, 95 % CI 0.98–1.06) (Fig. 2 and Supplementary Table 2). The dose-response relationship observed between UTC risk and radiation exposure (PC1) fitted with natural cubic splines was shown in Fig. 2 and Supplementary Table S3.

#### 3.1.2. Sensitivity analyses

HRs and 95 % CIs for different populations (all, HQ region, urban and rural), regression models (Model 1–3), sexes (total, men and women), and cancer types (UTC, kidney cancer, bladder cancer) were shown in Supplementary Figure S5, while results with FDR < 0.05 were annotated. For the samples who mainly (≥10 years) lived in regions with more measurements of radiation exposures (HQ region), a stronger association between radiation exposure and UTC risk was observed (Model 3: HR 1.10, 95 % CI 1.03–1.18). Similar patterns were observed among men (Model 3: HR 1.09, 95 % CI 1.00–1.18) and women (Model 3: HR 1.12, 95 % CI 1.00–1.25). When the total sample was stratified by years lived in an urban region, a stronger association between radiation exposure and UTC risk was observed in the rural sample (Model 3: HR 1.08, 95 % CI 1.02–1.14) than in the urban sample (Model 3: HR 1.03, 95 % CI 1.01–1.06). Model 1 and Model 2 found stronger associations between radiation exposure and UTC risk than Model 3 among all study populations. Patterns similar to those found in the main analyses were observed among men and women. Similar patterns were observed between radiation exposure and kidney/bladder cancer risks.

Based on log(−log) survival plots and Schoenfeld’s residual plots, no obverse violation of the proportional hazards assumption was observed.

### 3.2. UTC incidence and population attributable fraction

Age-standardized incidence rates (ASRs) of UTC per 100,000 person-years across exposure quintiles were tabulated in Table 2. In the total population, increasing UTC incidence was observed across exposure quintiles (lowest quintile [Q1] ASR=47, 95 % CI 45–50; highest quintile [Q5] ASR= 53, 95 % CI 50–55). Higher ASRs were observed among men than among women (Men: Q1 ASR=70, 95 % CI 65–75; Q5 ASR=86, 95 % CI 80–92; Women: Q1 ASR=30, 95 % CI 28–33; Q5 ASR=31, 95% CI 28–34). About 5.1 % of UTC incidence was attributable to radiation exposure in the total population (PAF=5.1 %, 95 % CI 0.5 %–9.7 %). About 9 % of UTC incidence was attributable to radiation exposure among men (PAF=9.0 %, 95 % CI 3.2 %–14.8 %). No significant PAF was observed among women (Table 2). For the samples who mainly (≥10 years) lived in regions with more measurements of radiation exposures (HQ region), 9.6 % of UTC incidence was attributable to radiation exposure (PAF=9.6 %, 95 % CI 3.3 %–16.0 %), and stronger patterns were observed in men and women (Men: PAF=12.9 %, 95 % CI 4.9 %–21.0 %; Women: PAF=10.0 %, 95 % CI −0.9 %–20.9 %) (Supplementary Table S4).

### 3.3. Regional patterns of environmental radiation and regional UTC risks

**Fig. 3.**
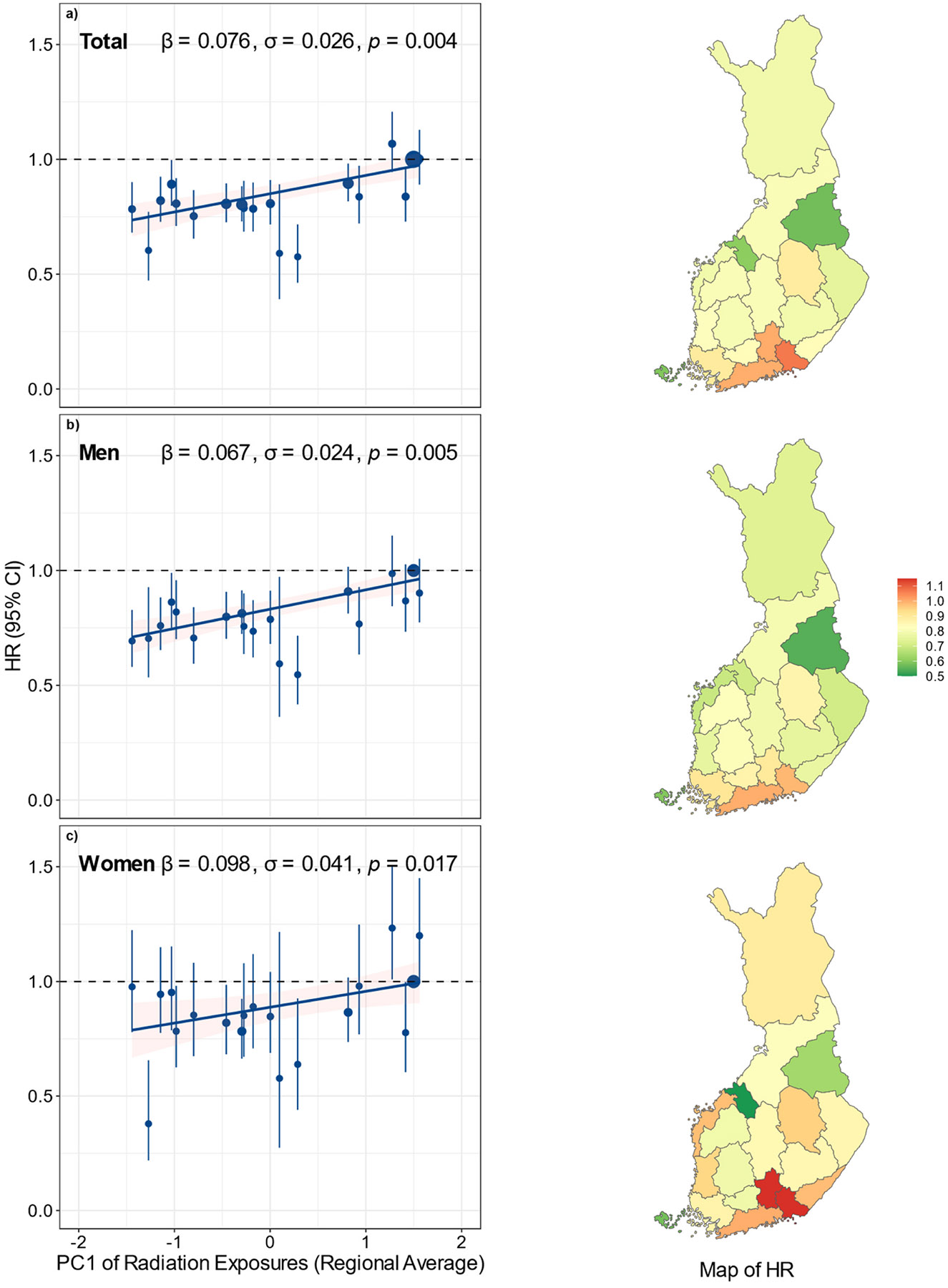
Spatial association between UTC risk (hazard ratio) and regional radiation exposure (PC1), Relationship between regional radiation level and regional UTC risk for a) the total population and for b) men and c) women. The error bars with dots are regional hazard ratios (HRs) and 95 % confidence intervals (CIs) of UTC incidence in 18 regions, using Uusimaa as the reference region (dot without error bar), estimated via Cox proportional hazards regression models using age as the time scale. *β* is the regression coefficient of PC1 (standardized) on UTC hazard ratios, *σ* is standard deviation, and *p* is statistical *p*-value. The maps on the right panel presented regional HRs. Regional exposure level was defined as the average of the first principal component (PC1) of municipality-level radiation exposures within each region. The size of the dots corresponded to the number of subjects in each region. The relationship between regional radiation level and the regional UTC risk was fitted using a linear spatial lag model.

Higher UTC risks were observed in southern Finland around Uusimaa for the total population and for men and women (Fig. 3, Supplementary Table S5). Regional UTC risk was significantly associated with regional radiation exposure adjusted for the spatial lag effect in the total population (*β* = 0.08, *p* < 0.01). A significant association was observed in both men (*β* = 0.07, *p* < 0.01) and women (*β* = 0.1, *p* = 0.02).

## 4. Discussion

In this population-based study, we tracked the residential locations of 2.8 million individuals living in Finland between 1987 until 2016, and linked them to long-term municipality-level environmental radioactivity data. We observed an association between increased UTC risk and high radiation exposure that was independent of birth cohort, socio-demographic factors, municipality-level lung cancer incidence rates, and family history of cancer and health conditions. A one standard deviation increase in the first principal component (PC1) of the three radiation exposures was associated with a 5 % higher UTC risk in the total population. A dose-response relationship was observed when the exposures were modelled as splines. In our study, consistent patterns were observed in samples who lived mainly (≥15 years) in a rural regions or urban regions. Stronger patterns were observed in samples who lived mainly (≥10 years) in municipalities with more radiation exposure measurements (HQ region).

As uranium and radon are classified as Group 1 human carcinogens, guidelines on their concentrations in environment and mitigation have been suggested and implemented by the WHO and by many countries, including Finland WHO [9,10,28,31]. The renal toxicity of uranium has been investigated in both animals and humans [34-38]. Uranium in the human body is eliminated rapidly via urine, and about 12–25 % is distributed and stored in the kidneys [39]. Positive associations between radiation exposure and kidney or bladder cancer mortalities were observed in uranium miners and uranium processing workers in France, Germany, and Canada [13,20]; in radiation workers in France, the UK, and the US [15,23]; and in a population living near uranium milling and mining operations in New Mexico [40]. Associations between radiation exposure and kidney and bladder cancer were reported in residents of Ukraine after the Chernobyl nuclear power plant accident, and in the general populations of the UK and Germany [11,14,41]. Many of these cohort studies included only highly selected exposed populations, had small sample sizes, and lacked general population controls.

However, the findings of previous epidemiological studies on the relationship between radiation exposure and UTCs were mixed [27,42]. Non-significant associations were reported in uranium miners from France, Germany, Canada, and the Czech Republic [19,21,22,43]. No significant association between radiation exposure and kidney cancer or bladder cancer was found in atomic bomb survivors in Japan, or in the general population of the US or Finland [16,18,25,26]. In observational studies of the general population, common concerns included inaccurate exposure assessments on the individual level and difficulties in tracking population movements and relocations over time [27].

In this study, we found that the UTC incidence rate increased across exposure quintile levels. About 5 % of UTC incidence was attributed to higher radiation exposure in the total population, while 9 % was attributed to higher radiation exposure in men, and no significant PAF was observed in women. Among the samples who mainly (≥10 years) lived in HQ regions, about 10 % of UTC incidence was attributable to higher radiation exposure, while 13 % was observed in men and 10 % in women. The fraction of UTC risk attributable to radiation exposure was comparable to the fraction of kidney cancer (renal cell carcinoma) risk attributable to smoking in the US (9 %–12 %) [44]. The incidence rate of UTC was about 2–3 times higher in men than in women, which was consistent with previous epidemiological studies [2,5,7,45]. The gender gaps between the incidence rates and the effect sizes of particular risk factors have not been fully investigated, and hypotheses on the effects of hormones, genetic factors, and metabolic mechanisms have been proposed [46-48].

The current well known risk factors of kidney/bladder cancer could not explain the geographical variation of UTC incidence in European countries [7,8]. Geographical variation of somatic mutation loads and patterns in kidney cancer (clear cell renal cell carcinoma) were reported in Eastern and Central Europe [49]. In this study, positive correlations were observed between regional radiation levels and regional UTC risks in the total population and in men and women. The results indicated that the geographical pattern of UTC could be partly explained by the pattern of environmental radiation exposures. In summary, this study systematically investigated the association between radiation exposure and UTC risk, providing insights that may be useful for cancer prevention and radiation protection strategies.

To our knowledge, this is the first study based on the total population of one country to evaluate the effects of long-term environmental radiation exposure on UTC risk and to examine their geographical correlations. The strengths of the present study include the large sample size drawn from the general population and the relatively accurate measurements of long-term radiation exposure that considered the movement and relocation of individuals over time. The data on uranium and radon concentrations in borehole waters were based on long-term measurements from the late 1970s to 2012 [28]. The data on concentrations of radon in indoor air were based on long-term measurements in residential buildings from 1986 to 2020 [30]. Given the potential unobserved confounding factors before and during the exposure period, such as radiation protection approaches introduced by STUK, the models were fitted conditional on five-year birth cohorts. While this approach could be considered over-adjusted and might lead to non-significant results, the association remained significant and demonstrated high robustness. Furthermore, the association was evaluated not only on the individual level, but also by incidence rates and PAF across exposure quintiles, and by spatial patterns across geographical regions.

This study had several limitations. First, we could only track annual exposures from water and indoor air at the municipality level for each individual, as there was no better measurement tool for the general population. Second, the uranium/radon levels we used in our study were proxies for environmental radioactivity levels, as there are other radiation elements that belong to the same decay families or that tend to occur in the same mineral deposits. We were unable to directly measure individual radioactivity exposure levels. Third, there was no information on occupational exposures. Fourth, the information on health conditions was based on data from registries on medical care and purchases of prescription medication. There might be a strong underestimation of health conditions such as obesity. Finally, there was no direct information in the national registries on lifestyle behaviours. We used municipality-level lung cancer incidence rates as proxies for the regional prevalence of smoking, but other factors, such as alcohol consumption, remained uncontrolled.

## 5. Conclusion

This population-based study provided robust evidence that long-term exposure to environmental radioactivity may be associated with increased UTC (kidney or bladder cancer) risks in the general population. Dose-response patterns were observed, especially among men. A small but notable proportion of UTC incidence was attributed to radiation exposure in the total population, while a higher proportion was observed among samples mainly lived in regions with more radiation exposure measurements. The geographical patterns of UTC (kidney or bladder cancer) incidence reported in epidemiological studies may be partly explained by long-term exposure to environmental radioactivity. Further investigations using direct measurements of individual radioactivity exposure levels are needed. Moreover, long-term observations and surveillance tools for monitoring radioactivity in specific regions might be necessary.

## Supplementary Material

1

2

## Figures and Tables

**Table 1:** Characteristics of socio-demographic factors, radiation exposures, and health conditions at baseline, and characteristics of UTC cancer diagnoses during the follow-up period for the total population and for men and women separately.

	Total N = 2,816,495	Men N = 1,368,777	Women N = 1,447,718
**Characteristics at baseline (Jan. 1, 2017)**			
**Age (years), mean (SD)**	60.1 (12.2)	59.0 (11.6)	61.2 (12.7)
**Uranium in water (μg/L), mean (SD)** ^a^	3.20 (1.50)	3.20 (1.50)	3.20 (1.50)
**Radon in water (Bq/L), mean (SD)** ^a^	6.22 (1.05)	6.20 (1.05)	6.24 (1.04)
**Radon in indoor air (Bq/m^3^), mean (SD)** ^a^	4.51 (0.46)	4.51 (0.46)	4.51 (0.46)
**PC1 of three exposures, mean (SD)** ^b^	0.00 (1.00)	−0.02 (1.00)	0.02 (1.00)
**Birth cohort** ^c^			
(1890,1935]	128,888 (4.6)	41,008 (3.0)	87,880 (6.1)
(1935,1945]	294,982 (10.5)	125,082 (9.1)	169,900 (11.7)
(1945,1955]	579,277 (20.6)	277,748 (20.3)	301,529 (20.8)
(1955,1965]	606,328 (21.5)	305,839 (22.3)	300,489 (20.8)
(1965,1975]	539,092 (19.1)	276,123 (20.2)	262,969 (18.2)
(1975,1985]	561,102 (19.9)	288,177 (21.1)	272,925 (18.9)
(1985,2000]	106,826 (3.8)	54,800 (4.0)	52,026 (3.6)
**Residential region at baseline, n (%)** ^d^			
Uusimaa	764,038 (27.1)	363,463 (26.6)	400,575 (27.7)
Varsinais-Suomi	245,358 (8.7)	117,810 (8.6)	127,548 (8.8)
Pirkanmaa	265,184 (9.4)	128,723 (9.4)	136,461 (9.4)
North Ostrobothnia	207,805 (7.4)	103,650 (7.6)	104,155 (7.2)
Others	1,334,110 (47.4)	655,131 (47.9)	678,979 (46.9)
**Urbanization level of residential municipality, n (%)**			
Urban	1,920,340 (68.2)	920,500 (67.3)	999,840 (69.1)
Semi-urban	476,071 (16.9)	235,373 (17.2)	240,698 (16.6)
Rural	420,084 (14.9)	212,904 (15.5)	207,180 (14.3)
**Years lived in urban areas, mean (SD)**	18.6 (12.3)	18.2 (12.4)	18.9 (12.2)
**Years lived in houses, mean (SD)**	19.5 (10.5)	20.0 (10.2)	19.0 (10.6)
**Years lived in HQ regions, mean (SD)** ^e^	15.3 (14.0)	15.1 (14.0)	15.4 (13.9)
**Marital status, n (%)**			
Married	1,495,946 (53.1)	749,910 (54.8)	746,036 (51.5)
Unmarried	710,514 (25.2)	399,776 (29.2)	310,738 (21.4)
Divorced	424,386 (15.1)	183,390 (13.4)	240,996 (16.7)
Widowed	185,649 (6.6)	35,701 (2.6)	149,948 (10.4)
**Education, n (%)**			
Low	595,979 (21.2)	297,151 (21.7)	298,828 (20.6)
Medium	1,172,575 (41.6)	621,507 (45.4)	551,068 (38.1)
High	1,047,941 (37.2)	450,119 (32.9)	597,822 (41.3)
**Income, n (%)**			
Low	903,658 (32.1)	401,169 (29.3)	502,489 (34.7)
Medium	785,388 (27.9)	384,627 (28.1)	400,761 (27.7)
High	1,127,449 (40.0)	582,981 (42.6)	544,468 (37.6)
**Health conditions, n (%)** ^f^			
Family history of cancer	997,857 (35.4)	510,192 (37.3)	487,665 (33.7)
Hypertension	411,520 (14.6)	194,106 (14.2)	217,414 (15.0)
Diabetes	293,018 (10.4)	151,982 (11.1)	141,036 (9.7)
Obesity	48,479 (1.7)	15,935 (1.2)	32,544 (2.3)
Chronic kidney disease	14,330 (0.5)	9587 (0.7)	4743 (0.3)
Calculus in kidney	44,526 (1.6)	29,353 (2.1)	15,173 (1.1)
Calculus in lower urinary tract	2889 (0.1)	2104 (0.2)	785 (0.1)
Urinary tract infections	63,538 (2.3)	8659 (0.6)	54,879 (3.8)
**Characteristics during follow-up period (from Jan. 1, 2017 to Dec. 31, 2021)**			
**Follow-up years, mean (SD)**	4.92 (0.49)	4.91 (0.51)	4.93 (0.45)
**Newly diagnosed urinary tract cancers, N (%in cancer cases)** ^g^			
UTC (C64–C68)	6718 (100)	4418 (100)	2300 (100)
Kidney (C64–C66, C68)	4462 (66.4)	2731 (61.8)	1731 (75.3)
Bladder (C67)	2256 (33.6)	1687 (38.2)	569 (24.7)

a The 30-year average radiation exposure was transformed to the natural logarithm scale. Samples with missing information on exposure to uranium in water or radon in water were excluded to calculate the mean (SD) in this table.

b The three 30-year average radiation exposures were transformed to the natural logarithm scale. Samples with missing information on exposure to uranium in water or radon in water were imputed with the sample mean. Principal component analyses were performed for the three exposures. The first principal component (PC1) was standardized and used as the proxy for environmental radiation exposures.

c Birth cohorts were stratified into 10-year groups in this table. Five-year birth cohorts were used in the analyses.

d A total of 19 regions in Finland were used in the analyses. The four regions with the largest population sizes were listed here.

e Region consisted of 79 municipalities with more measurements of the three radiation exposures.

f Proportion of samples with the corresponding health condition. The health conditions were not mutually exclusive.

g Only the primary cancer diagnosis was considered in this study. Samples diagnosed with other cancer types were censored at the date of diagnosis.

**Table 2:** Age-standardized incidence rate (ASR) of UTC across radiation exposure (PC1) quintiles and population attributable fraction (PAF) in the total population and stratified by sex.

Population	Exposure level quintiles^a^	Exposed person- years^b^	UTC cases^c^	ASR^d^ per 100,000 person- years	PAF (%) (95 % CI)
Total	Q1	2,777,584	1445	47 (45–50)	5.1 (0.5–9.7)*
	Q2	2,771,436	1312	48 (45–51)	
	Q3	2,779,443	1212	47 (44–50)	
	Q4	2,782,907	1339	54 (51–57)	
	Q5	2,778,532	1410	53 (50–55)	
Men	Q1	1,377,999	934	70 (65–75)	9.0 (3.2–14.8)**
	Q2	1,360,980	874	72 (67–77)	
	Q3	1,354,006	808	74 (69–80)	
	Q4	1,333,057	868	83 (77–89)	
	Q5	1,313,531	934	86 (80–92)	
Women	Q1	1,399,584	511	30 (28–33)	0.7 (−7.3–8.6)
	Q2	1,410,456	438	29 (27–32)	
	Q3	1,425,436	404	28 (25–31)	
	Q4	1,449,851	471	34 (31–37)	
	Q5	1,465,004	476	31 (28–34)	

Age-standardized incidence rate of UTC across radiation exposure (PC1) quintiles and population attributable fraction (PAF) of UTC risk associated with radiation exposure (PC1) in the total population and stratified by sex.

† *p*-value < 0.1

* *p*-value < 0.05

*** *p*-value < 0.001.

a First principal component (PC1) of 30-year average radiation exposures (uranium in water, radon in water, and radon in indoor air) calculated in the total study population was used as the proxy for radiation exposure. PC1 was stratified into quintiles in the total population.

b Person-years were calculated from the baseline until the earliest of the following events: diagnosis of primary urinary tract cancer, European standard population (2013 edition).

## Data Availability

Due to data protection regulations of the national register holders providing the data, we are not allowed to make the data available to third parties. Interested researchers have the option to request data access by contacting the following register-holding public institutions: Statistics Finland (http://www.stat.fi/tup/mikroaineistot/index_en.html). Contact by tutkijapalvelut(at)stat.fi. Findata (https://findata.fi/en/). Contact by info(at)findata.fi.

## Appendix A. Supporting information

Supplementary data associated with this article can be found in the online version at doi:10.1016/j.canep.2025.102912.
