# Supplementary material for: Long-term environmental background radiation is associated with urinary tract cancer incidence: A population-based study from Finland
Source: Cancer Epidemiol. Author manuscript; Available in PMC 2026 Jul 5. (PMC13333191; doi:10.1016/j.canep.2025.102912)
Supplement: 1 [file NIHMS2182287-supplement-1.docx]

Supplementary Figures

[Supplementary Figure S1: Maps and histograms of municipality-level radiation exposures 2](#_Toc202333022)

[Supplementary Figure S2: Maps and histograms of number of municipality-level radiation exposures 3](#_Toc202333023)

[Supplementary Figure S3: Scanter plots and principle component analyses of municipality-level radiation exposures 4](#_Toc202333024)

[Supplementary Figure S4: Histogram plots and principle component analyses of individual-level 30-year average radiation exposures 5](#_Toc202333025)

[Supplementary Figure S5: Sensitivity analyses of associations between UTC (kidney cancer, bladder cancer) and radiation exposure (PC1) 6](#_Toc202333026)

Supplementary Figure S1: Maps and histograms of municipality-level radiation exposures


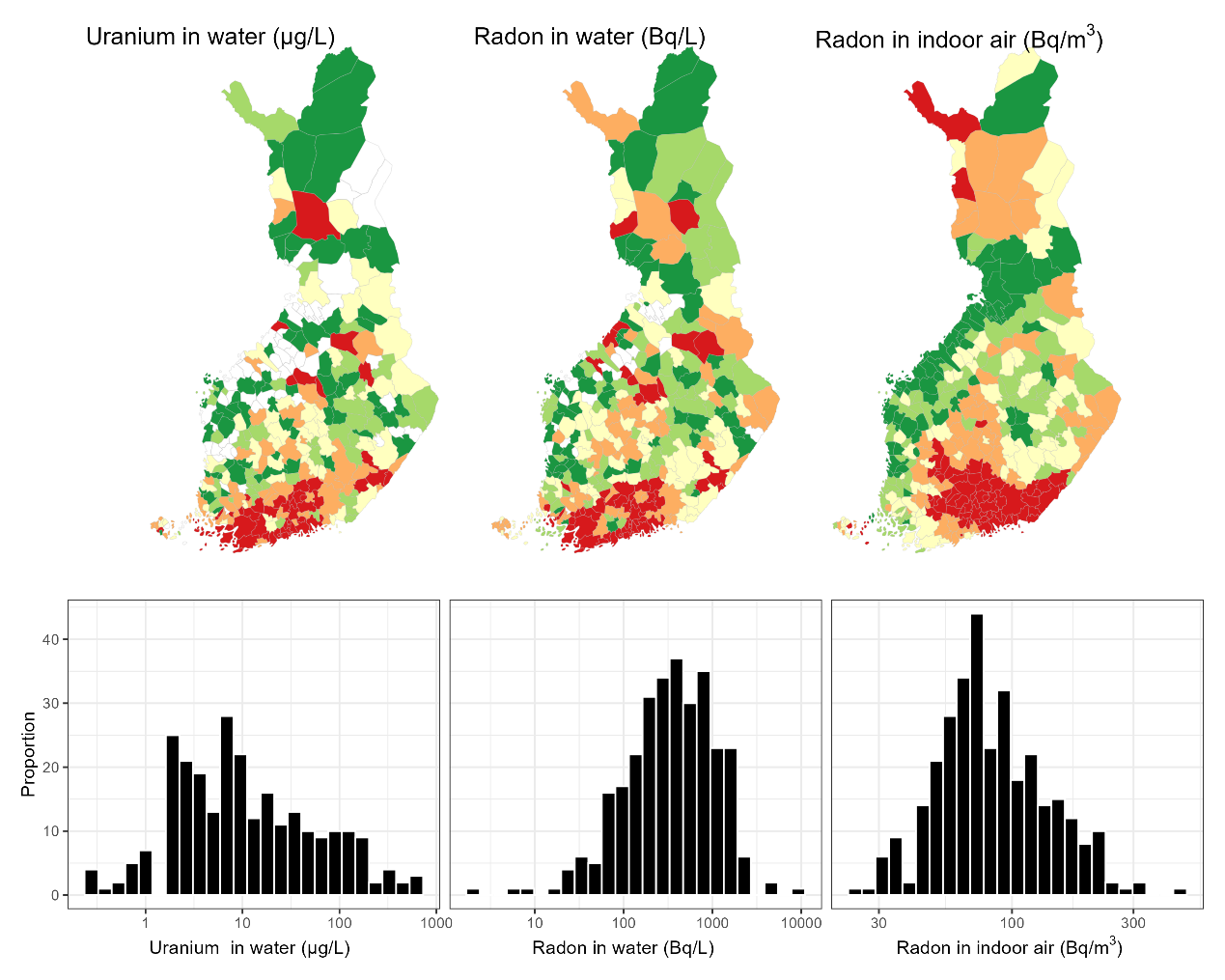


Upper panel a): The exposure levels of each municipality were classified into quintile groups for three radiation sources correspondingly: very low (dark green), low (green), medium (yellow), high (orange), and very high (red). b): The histograms of municipality-level exposures (log transformed).

Supplementary Figure S2: Maps and histograms of number of municipality-level radiation exposures


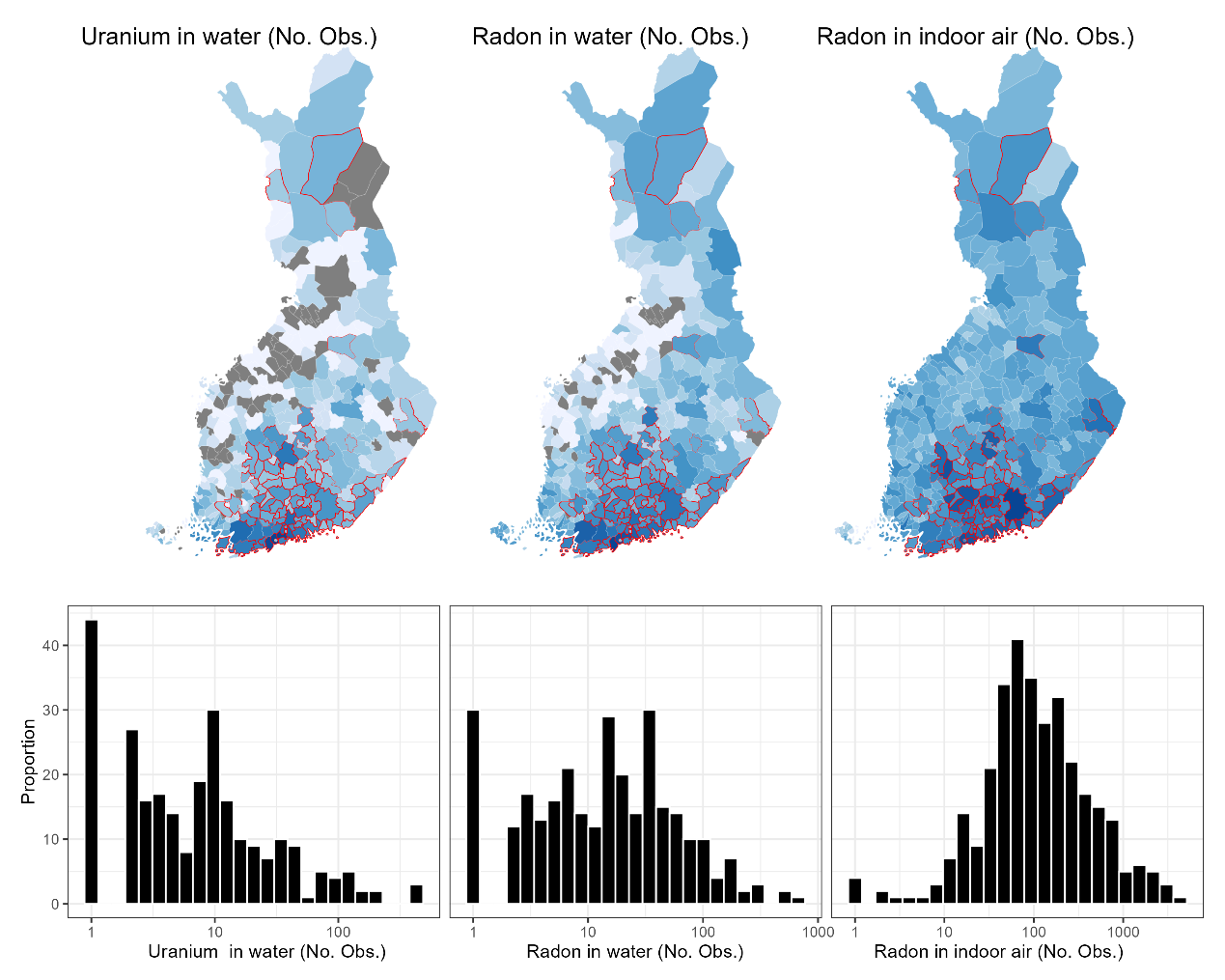


Upper panel: The maps of number of measurements (log transformed), municipalities with larger number of observations had darker colour. Totally 79 municipalities which had >5 measurements for uranium in water, >15 measurements for radon in water, >100 measurements for radon in indoor air and dwellings were measured in ≥5% of total residential buildings within each municipality were defined as region with high data quality (HQ-region) and highlighted with red boarder. Lower panel: The histograms of number of measurements of each municipality (log transformed).

Supplementary Figure S3: Scanter plots and principle component analyses of municipality-level radiation exposures


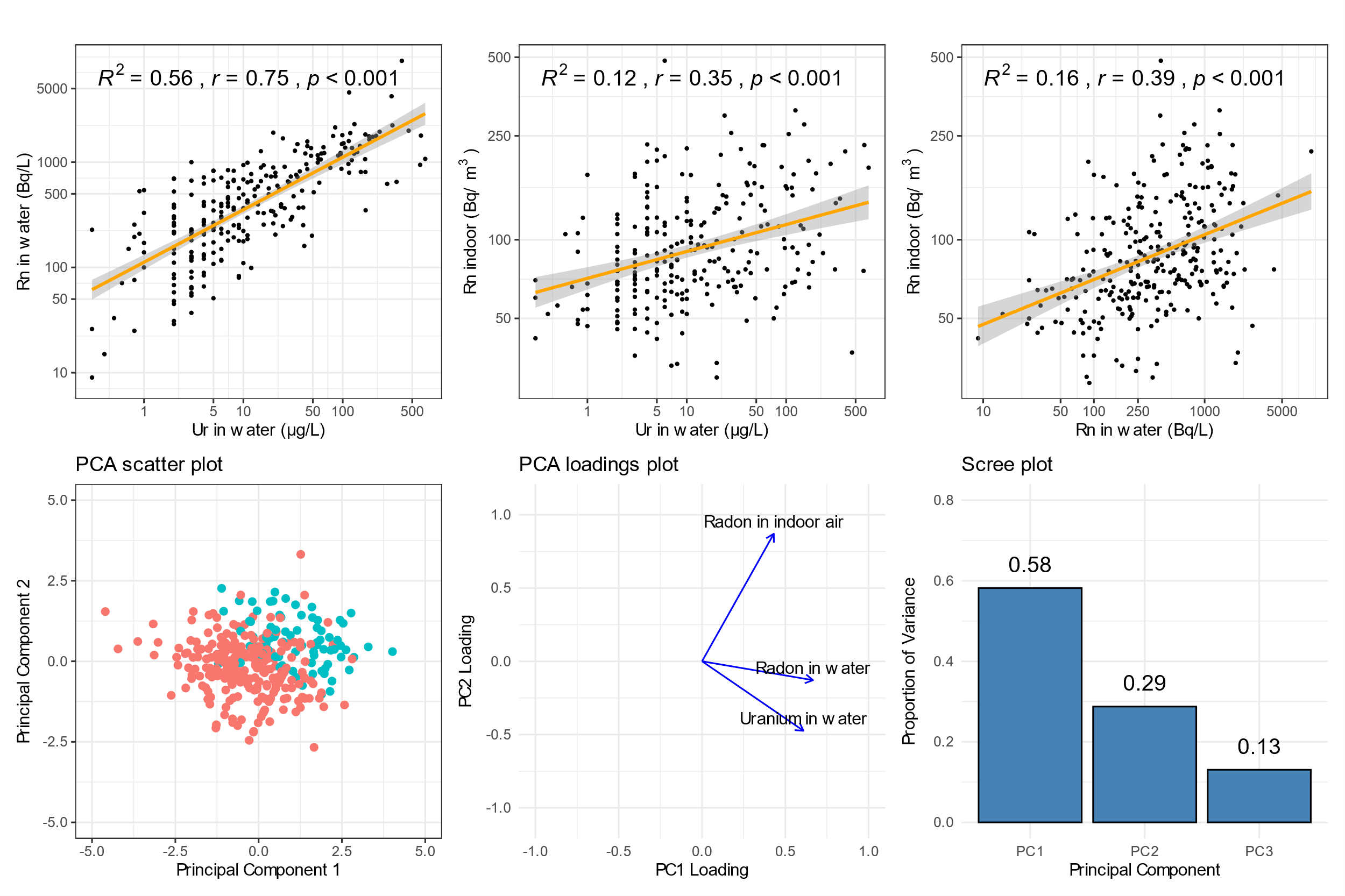


Upper panel: Each pair of exposures was shown in scatterplot in log-transformed scale and fitted via linear regression for all municipalities. The yellow line with shadow was the regression line and corresponding 95% confidence interval. The coefficient of determination (*R*^2^) and Pearson’s correlations coefficient (*r*) were annotated. Lower panel: Left: scant plot of first 2 principal components, where the blue dotes were HQ-region consisted municipalities with more measurements as defined in Supplementary Figure 2. Middle: principle component loadings plot for PC1 and PC2. Right: scree plot to show the proportion of variance explained by each principal component for all municipalities.

Supplementary Figure S4: Histogram plots and principle component analyses of individual-level 30-year average radiation exposures


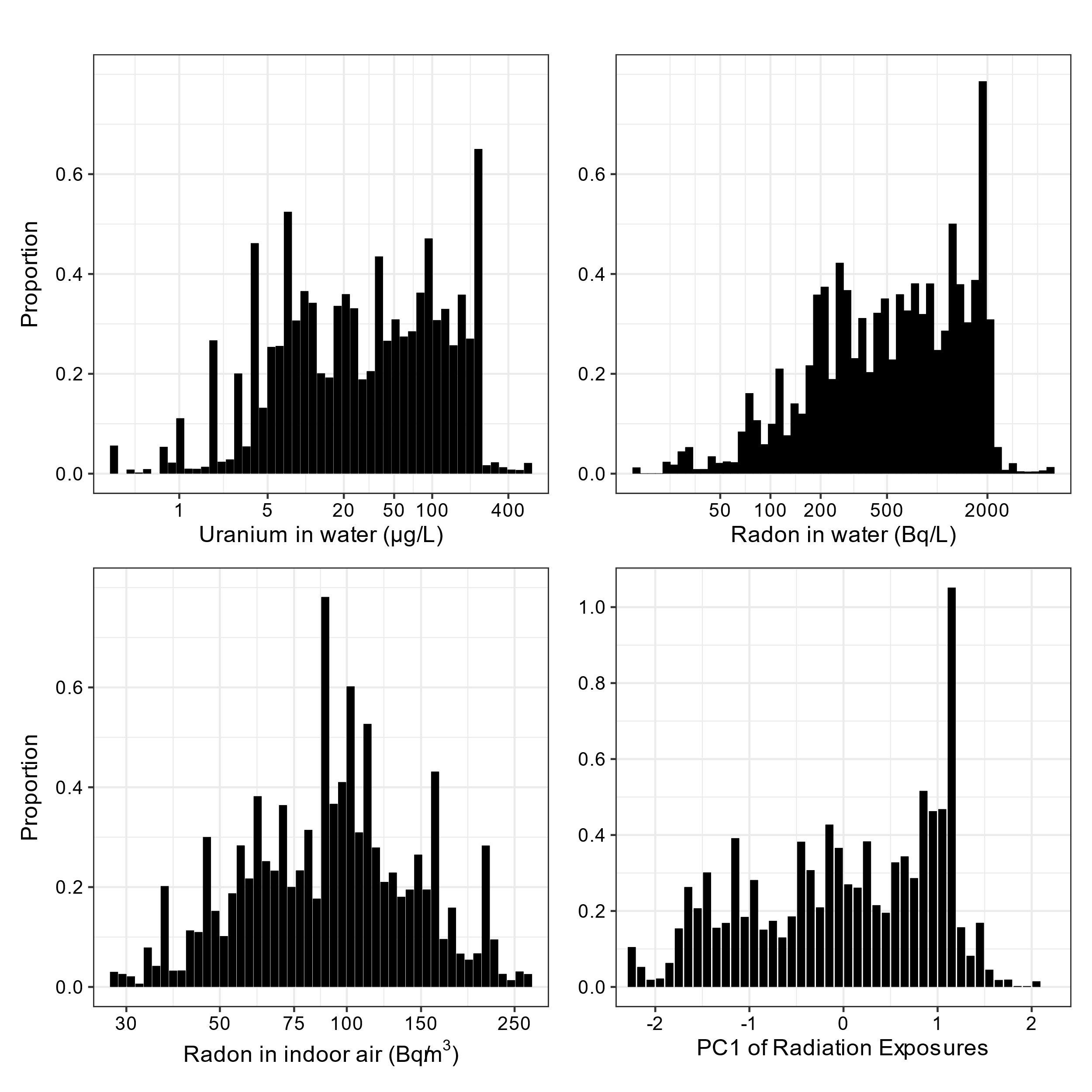


Histogram plots of individual-level 30-year average radiation exposures and first principal component (PC1) of three radiation exposures in total populaton.

Supplementary Figure S5: Sensitivity analyses of associations between UTC (kidney cancer, bladder cancer) and radiation exposure (PC1)


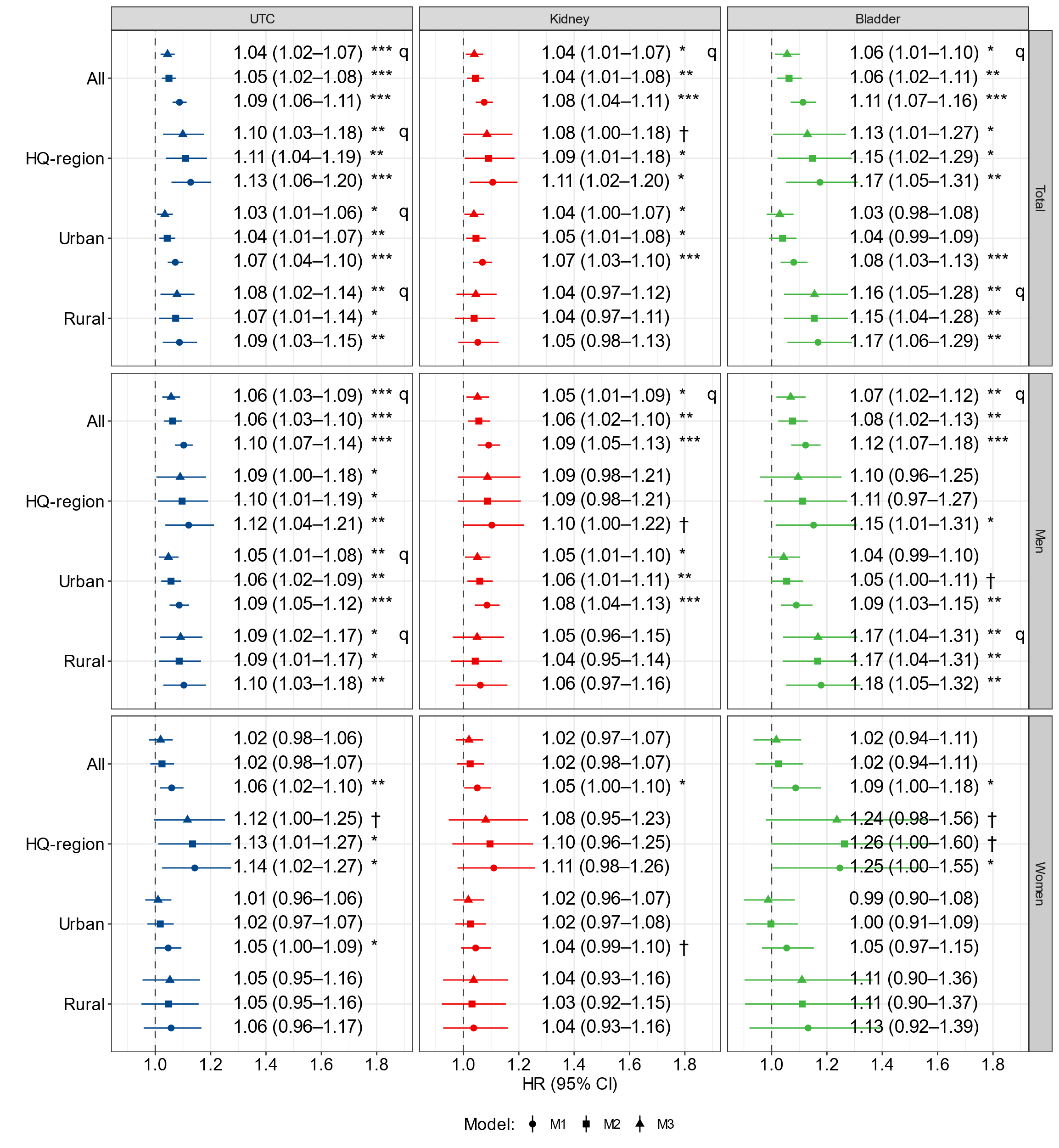


Hazard ratios (HRs) and 95% confidence intervals (CIs) of UTC (kidney cancer, bladder cancer) incidence for one SD increase of PC1 (first principle component of three radiation exposures) for different study population. "All sample" was the total population for both men and women, or stratified by men and women. "HQ-region sample" was the population who lived in HQ-region (as highlighted in Supplementary Figure 2) for ≥10 years. "Urban sample" was defined as population who lived in urban area for 15 years or more, while "rural sample" was population who lived in urban area less than 15 years. Model 1 was Cox proportional hazards regression model using age as the time scale, adjusted for principle components (PC1, PC2 and PC3) of three radiation exposures and sex for total sample. Model 2 was based Model 1 and further adjusted for individual education level, income, marital status, years lived in a house, years lived in an urban region, municipality-level lung cancer incidence rates, and health conditions (diabetes, hypertension, obesity, chronic kidney disease, calculus in kidney, calculus in lower urinary tract, and urinary tract infections) at baseline. Model 3 was based on Model 2 and conditional on 5-year birth cohorts. The same analyses were conducted stratified by sex. The tests annotated with "q" were with FDR < 0.05 across all the tests of M3.

† *p*-value < 0.1; * *p*-value < 0.05; ** *p*-value < 0.01; *** *p*-value < 0.001
