# Supplementary material for: Long-term environmental background radiation is associated with urinary tract cancer incidence: A population-based study from Finland
Source: Cancer Epidemiol. Author manuscript; Available in PMC 2026 Jul 5. (PMC13333191; doi:10.1016/j.canep.2025.102912)
Supplement: 2 [file NIHMS2182287-supplement-2.docx]

Supplementary Tables:

[Supplementary Table S1: Characteristics of socio-demographic factors and health conditions at baseline and characteristics of UTC cancer diagnoses during follow-up period across radiation exposure quintiles in total population 2](#_Toc206532476)

[Supplementary Table S2: Hazard ratios and 95% CIs of UTC incidence corresponding to radiation exposure and covariates 4](#_Toc206532477)

[Supplementary Table S3: Hazard ratios and 95% CIs of UTC incidence corresponding to radiation exposure and covariates 6](#_Toc206532478)

[Supplementary Table S4: Age-standards incidence rate (ASR) of UTC across radiation exposure (PC1) quintiles and population attributable fraction (PAF) in population mainly (≥10 years) lived in HQ-region and stratified by sex 8](#_Toc206532479)

[Supplementary Table S5: Regional exposure levels and hazard ratios and 95% CIs of UTC across regions 9](#_Toc206532480)

### Supplementary Table S1: Characteristics of socio-demographic factors and health conditions at baseline and characteristics of UTC cancer diagnoses during follow-up period across radiation exposure quintiles in total population

|  | **Q1^a^** | | **Q2^a^** | | **Q3^a^** | | **Q4^a^** | **Q5^a^** |
| --- | --- | --- | --- | --- | --- | --- | --- | --- |
|  | N=564,377 | | N=562,221 | | N=563,299 | | N=563,299 | N=563,299 |
| **Characteristics at baseline (1^st^ Jan 2017)** | |  | | | |  | | |
| **Sex** |  | |  | |  | |  |  |
| Men | 280,335 (49.67%) | | 276,496 (49.18%) | | 274,883 (48.80%) | | 270,320 (47.99%) | 266,743 (47.35%) |
| Women | 284,042 (50.33%) | | 285,725 (50.82%) | | 288,416 (51.20%) | | 292,979 (52.01%) | 296,556 (52.65%) |
| **Age (years), mean (SD)** | 56.56 (15.32) | | 54.62 (15.46) | | 53.68 (15.33) | | 53.40 (14.84) | 54.66 (14.77) |
| **Birth cohort ^b^** | |  | | | |  | | |
| (1890,1935] | 32860 (5.83%) | | 27731 (4.93%) | | 24960 (4.43%) | | 20845 (3.70%) | 22492 (3.99%) |
| (1935,1945] | 67,921 (12.03%) | | 59,333 (10.55%) | | 55,590 (9.87%) | | 53,546 (9.51%) | 58,592 (10.40%) |
| (1945,1955] | 131,125 (23.23%) | | 116,567 (20.73%) | | 108,034 (19.18%) | | 106,544 (18.91%) | 117,007 (20.77%) |
| (1955,1965] | 124,852 (22.12%) | | 118,101 (21.01%) | | 116,595 (20.70%) | | 118,934 (21.11%) | 127,846 (22.70%) |
| (1965,1975] | 93,776 (16.62%) | | 102,476 (18.23%) | | 109,903 (19.51%) | | 120,535 (21.40%) | 112,402 (19.95%) |
| (1975,1985] | 95,196 (16.87%) | | 114,837 (20.43%) | | 124,426 (22.09%) | | 121,519 (21.57%) | 105,124 (18.66%) |
| (1985,2000] | 18,647 (3.30%) | | 23,176 (4.12%) | | 23,791 (4.22%) | | 21,376 (3.79%) | 19,836 (3.52%) |
| **Residential region at baseline, n (%) ^c^** | | | |  | | | | |
| Uusimaa | 1,259 (0.22%) | | 27,122 (4.82%) | | 53,934 (9.57%) | | 258,768 (45.94%) | 422,955 (75.09%) |
| Varsinais-Suomi | 14,283 (2.53%) | | 96,434 (17.15%) | | 69,869 (12.40%) | | 58,089 (10.31%) | 6,683 (1.19%) |
| Pirkanmaa | 20,452 (3.62%) | | 77,027 (13.70%) | | 146,322 (25.98%) | | 17,270 (3.07%) | 4,113 (0.73%) |
| North Ostrobothnia | 139,507 (24.72%) | | 42,184 (7.50%) | | 19,723 (3.50%) | | 5,594 (0.99%) | 797 (0.14%) |
| Others | 388,876 (68.90%) | | 319,454 (56.82%) | | 273,451 (48.54%) | | 223,578 (39.69%) | 128,751 (22.86%) |
| **Urbanisation level of residential municipality, n (%)** | | | |  | | | | |
| Urban | 326,072 (57.78%) | | 278,289 (49.50%) | | 363,219 (64.48%) | | 479,556 (85.13%) | 473,204 (84.01%) |
| Semi-urban | 122,875 (21.77%) | | 151,850 (27.01%) | | 107,309 (19.05%) | | 42,911 (7.62%) | 51,126 (9.08%) |
| Rural | 115,430 (20.45%) | | 132,082 (23.49%) | | 92,771 (16.47%) | | 40,832 (7.25%) | 38,969 (6.92%) |
| **Years lived in urban areas, mean (SD)** | 326,072 (57.78%) | | 278,289 (49.50%) | | 363,219 (64.48%) | | 479,556 (85.13%) | 473,204 (84.01%) |
| **Years lived in houses, mean (SD)** | 23.21 (9.14) | | 21.74 (9.48) | | 20.11 (9.71) | | 18.49 (9.93) | 13.92 (11.30) |
| **Years live in HQ-region, mean (SD) ^d^** | 0.42 (3.06) | | 7.30 (11.34) | | 16.78 (12.88) | | 22.86 (10.92) | 28.94 (4.26) |
| **Marital status, n (%)** |  | |  | |  | |  |  |
| Married | 316,751 (56.12%) | | 302,263 (53.76%) | | 296,772 (52.68%) | | 298,138 (52.93%) | 282,022 (50.07%) |
| Unmarried | 126,167 (22.36%) | | 140,388 (24.97%) | | 146,055 (25.93%) | | 143,500 (25.47%) | 154,404 (27.41%) |
| Divorced | 76,077 (13.48%) | | 79,912 (14.21%) | | 84,629 (15.02%) | | 89,818 (15.94%) | 93,950 (16.68%) |
| Widowed | 45,382 (8.04%) | | 39,658 (7.05%) | | 35,843 (6.36%) | | 31,843 (5.65%) | 32,923 (5.84%) |
| **Education, n (%)** |  | |  | |  | |  |  |
| Low | 132,224 (23.43%) | | 123,564 (21.98%) | | 112,482 (19.97%) | | 105,831 (18.79%) | 121,878 (21.64%) |
| Medium | 259,704 (46.02%) | | 244,458 (43.48%) | | 238,559 (42.35%) | | 218,381 (38.77%) | 211,473 (37.54%) |
| High | 172,449 (30.56%) | | 194,199 (34.54%) | | 212,258 (37.68%) | | 239,087 (42.44%) | 229,948 (40.82%) |
| **Income, n (%)** |  | |  | |  | |  |  |
| Low | 207,013 (36.68%) | | 190,252 (33.84%) | | 179,042 (31.78%) | | 163,205 (28.97%) | 164,146 (29.14%) |
| Medium | 159,755 (28.31%) | | 164,179 (29.20%) | | 162,372 (28.83%) | | 155,117 (27.54%) | 143,965 (25.56%) |
| High | 197,609 (35.01%) | | 207,790 (36.96%) | | 221,885 (39.39%) | | 244,977 (43.49%) | 255,188 (45.30%) |
| **Health conditions, N (%) ^e^** |  | |  | |  | |  |  |
| Family history of cancer | 200,275 (35.49%) | | 195,819 (34.83%) | | 194,717 (34.57%) | | 201,288 (35.73%) | 205,758 (36.53%) |
| Hypertension | 102,795 (18.21%) | | 84,323 (15.00%) | | 78,311 (13.90%) | | 71,108 (12.62%) | 74,983 (13.31%) |
| Diabetes | 65,798 (11.66%) | | 60,409 (10.74%) | | 55,839 (9.91%) | | 54,164 (9.62%) | 56,808 (10.08%) |
| Obesity | 9,971 (1.77%) | | 10,416 (1.85%) | | 9,409 (1.67%) | | 9,214 (1.64%) | 9,469 (1.68%) |
| Chronic kidney disease | 3,169 (0.56%) | | 3,082 (0.55%) | | 2,834 (0.50%) | | 2,770 (0.49%) | 2,475 (0.44%) |
| Calculus in kidney | 9,626 (1.71%) | | 8,596 (1.53%) | | 8,814 (1.56%) | | 9,115 (1.62%) | 8,375 (1.49%) |
| Calculus in low urinary tract | 687 (0.12%) | | 581 (0.10%) | | 536 (0.10%) | | 509 (0.09%) | 576 (0.10%) |
| Urinary tract infections | 13,553 (2.40%) | | 12,519 (2.23%) | | 13,438 (2.39%) | | 12,993 (2.31%) | 11,035 (1.96%) |
| **Characteristics during follow-up period (from 1 Jan 2017 to 31 Dec 2021)** | | | | | | | | |
| **Follow-up years, mean (SD)** | 4.92 (0.47) | | 4.93 (0.44) | | 4.93 (0.43) | | 4.94 (0.41) | 4.93 (0.43) |
| **Newly diagnosed urinary tract cancers, N (% in cancer cases)** **^f^** | | | | | | | | |
| UTC (C64–C68) | 1,445 (0.26%) | | 1,312 (0.23%) | | 1,212 (0.22%) | | 1,339 (0.24%) | 1,410 (0.25%) |
| Kidney (C64–C66, C68) | 960 (66.44%) | | 879 (67.00%) | | 809 (66.75%) | | 901 (67.29%) | 913 (64.75%) |
| Bladder (C67) | 485 (33.56%) | | 433 (33.00%) | | 403 (33.25%) | | 438 (32.71%) | 497 (35.25%) |

^a^ First principal component (PC1) of three radiation exposures was stratified into quintiles.

^b^ Birth cohorts were stratified into 10-year groups in this table. Five-year birth cohorts were used in the analyses.

^c^ A total of 19 regions in Finland were used in the analyses. The four regions with the largest population sizes were listed here.

^d^ Municipalities with more measurements of three radiation exposures (HQ-region).

^e^ Proportion of samples with the corresponding health condition. The health conditions were not mutually exclusive.

^f^ Only the primary cancer diagnosis was considered. Samples diagnosed with other cancer types were censored at the date of diagnosis.

### Supplementary Table S2: Hazard ratios and 95% CIs of UTC incidence corresponding to radiation exposure and covariates

| **Factors** | **Total** | **Men** | **Women** |
| --- | --- | --- | --- |
| **Radiation exposure (PCA)** |  |  |  |
| PC1 | 1.04 (1.02–1.07)*** | 1.06 (1.03–1.09)*** | 1.02 (0.98–1.06) |
| PC2 | 1.00 (0.98–1.03) | 1.00 (0.97–1.03) | 1.01 (0.97–1.05) |
| PC3 | 1.00 (0.98–1.03) | 1.02 (0.99–1.05) | 0.98 (0.94–1.02) |
| **Sex** |  |  |  |
| Men | Ref |  | Ref |
| Women | 0.41 (0.39–0.43)*** |  |  |
| **Marital status** |  |  |  |
| Married | Ref | Ref | Ref |
| Unmarried | 0.96 (0.89–1.04) | 0.95 (0.86–1.04) | 1.06 (0.92–1.22) |
| Divorced | 0.99 (0.93–1.07) | 1.02 (0.94–1.12) | 0.98 (0.87–1.10) |
| Widowed | 0.96 (0.89–1.05) | 0.96 (0.85–1.09) | 1.03 (0.91–1.16) |
| **Education** |  |  |  |
| Low | Ref | Ref | Ref |
| Medium | 1.03 (0.97–1.09) | 1.02 (0.95–1.10) | 1.04 (0.94–1.15) |
| High | 0.91 (0.85–0.97)** | 0.92 (0.85–1.00)† | 0.85 (0.75–0.95)** |
| **Income** |  |  |  |
| Low | Ref | Ref | Ref |
| Medium | 0.99 (0.93–1.06) | 1.02 (0.94–1.11) | 0.95 (0.84–1.07) |
| High | 0.98 (0.91–1.05) | 1.00 (0.92–1.09) | 0.93 (0.82–1.06) |
| **Years lived in urban areas** | 1.00 (0.999–1.00) | 1.00 (0.999–1.01) | 1.00 (0.997–1.00) |
| **Years lived in houses** |  |  |  |
| ≤ 10 years | Ref | Ref | Ref |
| > 10 and ≤ 20 years | 0.94 (0.87–1.03) | 0.98 (0.89–1.08) | 0.88 (0.76–1.01)† |
| > 20 and ≤ 30 years | 0.86 (0.80–0.91)*** | 0.85 (0.79–0.92)*** | 0.86 (0.77–0.95)** |
| **Municipality-level ASR of lung cancer** | 1.00 (0.996–1.01) | 1.00 (0.99–1.01) | 1.00 (0.99–1.01) |
| **Health conditions** |  |  |  |
| Family history of cancer | 1.16 (1.09–1.23)*** | 1.12 (1.05–1.20)*** | 1.23 (1.11–1.37)*** |
| Hypertension | 1.33 (1.26–1.41)*** | 1.33 (1.24–1.42)*** | 1.37 (1.25–1.50)*** |
| Diabetes | 1.35 (1.28–1.44)*** | 1.35 (1.26–1.45)*** | 1.36 (1.22–1.50)*** |
| Obesity | 1.51 (1.28–1.78)*** | 1.42 (1.15–1.77)** | 1.62 (1.26–2.07)*** |
| Chronic kidney disease | 1.83 (1.56–2.15)*** | 1.67 (1.39–2.01)*** | 2.37 (1.70–3.31)*** |
| Calculus in kidney | 1.17 (1.02–1.34)* | 1.13 (0.98–1.32) | 1.30 (0.96–1.78)† |
| Calculus in low urinary tract | 1.29 (0.84–1.97) | 1.13 (0.70–1.81) | 2.39 (0.90–6.39)† |
| Urinary tract infections | 1.11 (0.97–1.28) | 1.29 (1.03–1.62)* | 1.05 (0.88–1.25) |

Hazard ratios (HRs) and 95% confidence intervals (CIs) of UTC incidence for different covariates. Cox proportional hazards regression model and conditional on 5-year birth cohorts and using age as the time scale, was adjusted for principle components (PC1, PC2 and PC3) of three radiation exposures and sex for total sample and further adjusted for individual education level, income, marital status, years lived in a house, years lived in an urban region, municipality-level lung cancer incidence rates, and health conditions (diabetes, hypertension, obesity, chronic kidney disease, calculus in kidney, calculus in lower urinary tract, and urinary tract infections) at baseline. The same analyses were conducted stratified by sex. Principle components of three radiation exposures (PC1, PC2 and PC3) were standardized.

† *p*-value < 0.1; * *p*-value < 0.05; *** *p*-value < 0.001

### Supplementary Table S3: Hazard ratios and 95% CIs of UTC incidence corresponding to radiation exposure and covariates

| **Factors** | **Total** | **Men** | **Women** |
| --- | --- | --- | --- |
| **Radiation exposure (PCA)** |  |  |  |
| PC1 (spline-1) | 1.88 (0.63–5.65) | 2.07 (0.52–8.25) | 1.62 (0.26–9.91) |
| PC1 (spline-2) | 1.14 (0.76–1.71) | 1.29 (0.78–2.13) | 0.92 (0.47–1.82) |
| PC1 (spline-3) | 2.01 (0.90–4.50)† | 2.23 (0.81–6.12) | 1.67 (0.44–6.38) |
| PC2 | 1.00 (0.98–1.03) | 1.00 (0.97–1.03) | 1.01 (0.97–1.05) |
| PC3 | 1.00 (0.98–1.03) | 1.02 (0.99–1.05) | 0.98 (0.94–1.02) |
| **Sex** |  |  |  |
| Men | Ref |  |  |
| Women | 0.41 (0.39–0.43)*** |  |  |
| **Marital status** |  |  |  |
| Married | Ref | Ref | Ref |
| Unmarried | 0.96 (0.89–1.04) | 0.95 (0.86–1.04) | 1.06 (0.92–1.22) |
| Divorced | 0.99 (0.93–1.07) | 1.02 (0.94–1.12) | 0.98 (0.87–1.10) |
| Widowed | 0.96 (0.89–1.05) | 0.96 (0.85–1.09) | 1.03 (0.91–1.16) |
| **Education** |  |  |  |
| Low | Ref | Ref | Ref |
| Medium | 1.03 (0.97–1.09) | 1.02 (0.95–1.10) | 1.04 (0.94–1.16) |
| High | 0.91 (0.85–0.97)** | 0.92 (0.85–1.00)† | 0.85 (0.75–0.95)** |
| **Income** |  |  |  |
| Low | Ref | Ref | Ref |
| Medium | 0.99 (0.93–1.06) | 1.02 (0.94–1.11) | 0.95 (0.84–1.07) |
| High | 0.98 (0.91–1.05) | 1.00 (0.92–1.09) | 0.93 (0.82–1.06) |
| **Years lived in urban areas** | 1.00 (0.999–1.00) | 1.00 (0.999–1.01) | 1.00 (0.997–1.00) |
| **Years lived in houses** |  |  |  |
| ≤ 10 years | Ref | Ref | Ref |
| ≥ 11 and ≤ 20 years | 0.95 (0.87–1.03) | 0.98 (0.89–1.08) | 0.88 (0.76–1.01)† |
| ≥ 21 and ≤ 30 years | 0.86 (0.80–0.91)*** | 0.85 (0.79–0.92)*** | 0.86 (0.77–0.95)** |
| **Municipality-level ASR of lung cancer** | 1.00 (0.996–1.01) | 1.00 (0.99–1.01) | 1.00 (0.99–1.01) |
| **Health conditions** |  |  |  |
| Family history of cancer | 1.16 (1.09–1.23)*** | 1.12 (1.05–1.20)*** | 1.23 (1.11–1.37)*** |
| Hypertension | 1.33 (1.26–1.41)*** | 1.33 (1.24–1.42)*** | 1.37 (1.25–1.50)*** |
| Diabetes | 1.35 (1.28–1.44)*** | 1.35 (1.26–1.45)*** | 1.36 (1.22–1.50)*** |
| Obesity | 1.51 (1.28–1.78)*** | 1.42 (1.15–1.77)** | 1.62 (1.26–2.07)*** |
| Chronic kidney disease | 1.83 (1.56–2.15)*** | 1.67 (1.39–2.01)*** | 2.37 (1.70–3.31)*** |
| Calculus in kidney | 1.17 (1.02–1.34)* | 1.13 (0.98–1.32) | 1.30 (0.96–1.78)† |
| Calculus in low urinary tract | 1.29 (0.84–1.97) | 1.12 (0.70–1.81) | 2.39 (0.90–6.39)† |
| Urinary tract infections | 1.11 (0.97–1.28) | 1.29 (1.03–1.62)* | 1.05 (0.88–1.25) |

Hazard ratios (HRs) and 95% confidence intervals (CIs) of UTC incidence for different covariates. Cox proportional hazards regression model and conditional on 5-year birth cohorts and using age as the time scale, was adjusted for principle components (PC1, PC2 and PC3) of three radiation exposures and sex for total sample and further adjusted for individual education level, income, marital status, years lived in a house, years lived in an urban region, municipality-level lung cancer incidence rates, and health conditions (diabetes, hypertension, obesity, chronic kidney disease, calculus in kidney, calculus in lower urinary tract, and urinary tract infections) at baseline. The same analyses were conducted stratified by sex. Principle components of three radiation exposures (PC1, PC2 and PC3) were standardized, and PC1 was modelled via 3-degree natural cubic splines.

† *p*-value < 0.1; * *p*-value < 0.05; *** *p*-value < 0.001

### Supplementary Table S4: Age-standards incidence rate (ASR) of UTC across radiation exposure (PC1) quintiles and population attributable fraction (PAF) in population mainly (≥10 years) lived in HQ-region and stratified by sex

| **Population** | **Exposure**  **level** **quintiles** ^a^ | **Exposed**  **person-years** ^b^ | **UTC cases** ^c^ | **ASR** ^d^  **per 100,000 person-years** | **PAF (%)**  **(95%CI)** |
| --- | --- | --- | --- | --- | --- |
| Total | Q1 | 1513245 | 688 | 46 (42–49) | 9.6 (3.3–16.0) ** |
|  | Q2 | 1515727 | 645 | 49 (39–46) |  |
|  | Q3 | 1547582 | 727 | 53 (44–50) |  |
|  | Q4 | 1488566 | 724 | 59 (45–52) |  |
|  | Q5 | 1508469 | 782 | 50 (48–55) |  |
| Men | Q1 | 743123 | 461 | 71 (56–68) | 12.9 (4.9–21.0)** |
|  | Q2 | 725943 | 424 | 81 (53–64) |  |
|  | Q3 | 737298 | 477 | 84 (59–71) |  |
|  | Q4 | 713213 | 482 | 92 (62–74) |  |
|  | Q5 | 704475 | 508 | 81 (66–78) |  |
| Women | Q1 | 770122 | 227 | 28 (26–33) | 10.0 (-0.9–20.9) † |
|  | Q2 | 789785 | 221 | 30 (24–32) |  |
|  | Q3 | 810285 | 250 | 33 (27–35) |  |
|  | Q4 | 775356 | 242 | 35 (27–35) |  |
|  | Q5 | 803995 | 274 | 30 (30–38) |  |

Age-standards incidence rate of UTC across radiation exposure (PC1) quintiles and population attributable fraction (PAF) of UTC risk associated with radiation exposure (PC1) in population mainly (≥10 years) lived in HQ-region and stratified by sex.

^a^ First principal component (PC1) of 30-year average radiation exposures (uranium in water, radon in water and radon in indoor air) calculated in total study population were used as the proxy of radiation exposure. PC1 was stratified into quintiles in total population.

^b^ Person-years were calculated from the baseline until the earliest of the following events: diagnosis of primary urinary tract cancer, other types of cancer, emigration, death, or the end of study.

^c^ Only primary UTC cases diagnosed during the follow-up period were considered in this study. Samples diagnosed with other cancer types were censored at the date of diagnosis.

^d^ ASRs of UTC per 100,000 person-years were reported for each quintile group using European standard population (2013 edition).

† *p*-value < 0.1; * *p*-value < 0.05; *** *p*-value < 0.001

### Supplementary Table S5: Regional exposure levels and hazard ratios and 95% CIs of UTC across regions

| **Region** | **Exposure**  **PC1** | **HR (95%CI)**  **Total** | **HR (95%CI)**  **Men** | **HR (95%CI)**  **Women** |
| --- | --- | --- | --- | --- |
| Uusimaa | 1.50 | Ref | Ref | Ref |
| Varsinais-Suomi | 0.81 | 0.90 (0.82–0.98)* | 0.91 (0.81–1.02)† | 0.87 (0.74–1.02)† |
| Satakunta | -1.15 | 0.82 (0.73–0.92)** | 0.76 (0.65–0.88)*** | 0.94 (0.78–1.15) |
| Kanta-Häme | 1.41 | 0.84 (0.73–0.96)* | 0.87 (0.73–1.03)† | 0.78 (0.60–1.00)* |
| Pirkanmaa | -0.30 | 0.80 (0.73–0.88)*** | 0.81 (0.72–0.91)*** | 0.78 (0.66–0.92)** |
| Päijät-Häme | 1.56 | 1.00 (0.89–1.13) | 0.90 (0.77–1.05) | 1.20 (0.99–1.45)† |
| Kymenlaakso | 1.27 | 1.07 (0.94–1.21) | 0.99 (0.84–1.15) | 1.23 (1.01–1.51)* |
| South Karelia | 0.93 | 0.84 (0.72–0.97)* | 0.77 (0.63–0.93)** | 0.98 (0.77–1.25) |
| Etelä-Savo | -0.27 | 0.79 (0.68–0.91)*** | 0.76 (0.64–0.90)** | 0.85 (0.67–1.08) |
| Pohjois-Savo | -1.03 | 0.89 (0.80–1.00)* | 0.86 (0.75–0.99)* | 0.95 (0.79–1.15) |
| North Karelia | -0.80 | 0.75 (0.65–0.87)*** | 0.71 (0.59–0.84)*** | 0.85 (0.67–1.08) |
| Central Finland | 0.00 | 0.81 (0.72–0.91)*** | 0.79 (0.68–0.91)** | 0.85 (0.69–1.04) |
| South Ostrobothnia | -0.98 | 0.81 (0.71–0.92)** | 0.82 (0.70–0.96)* | 0.78 (0.63–0.98)* |
| Ostrobothnia | -1.44 | 0.78 (0.68–0.90)*** | 0.69 (0.58–0.83)*** | 0.98 (0.78–1.22) |
| Central Ostrobothnia | -1.27 | 0.60 (0.47–0.77)*** | 0.70 (0.53–0.93)* | 0.38 (0.22–0.66)*** |
| North Ostrobothnia | -0.46 | 0.81 (0.73–0.90)*** | 0.80 (0.70–0.91)*** | 0.82 (0.68–0.99)* |
| Kainuu | 0.29 | 0.58 (0.46–0.72)*** | 0.55 (0.42–0.72)*** | 0.64 (0.44–0.93)* |
| Lapland | -0.18 | 0.78 (0.69–0.90)*** | 0.74 (0.62–0.87)*** | 0.89 (0.71–1.12) |
| Åland | 0.10 | 0.59 (0.39–0.89)* | 0.59 (0.36–0.97)* | 0.58 (0.27–1.22) |

Regional exposure levels (PC1) and hazard ratios (HRs) and 95% confidence intervals (CIs) of UTC incidence across regions using Uusimaa as reference region. The regional exposure level was defined as the average municipality-level exposures of municipalities within each region. All the Cox proportional hazards regression models used age as the time scale.

† *p*-value < 0.1; * *p*-value < 0.05; *** *p*-value < 0.001
